# Air Pollution and Corporate Green Financial Constraints: Evidence from China’s Listed Companies

**DOI:** 10.3390/ijerph192215034

**Published:** 2022-11-15

**Authors:** Yi Shen, Minghan Lyu, Jiali Zhu

**Affiliations:** 1 College of Economics and Management, Nanjing Forestry University, Nanjing 210037, China; 2 Shanghai National Accounting Institute, Shanghai 201702, China; 3 School of Economics and Management, Nanjing University of Science and Technology, Nanjing 210094, China

**Keywords:** air pollution, corporate green investment, financial constraints, institutional environment

## Abstract

This paper aims to investigate how air pollution may affect corporate green financial constraints. We assume that poor air quality can enhance the pressure of governments on environmental protection, which creates easier access to financing for firms’ green investments and transitions, especially in emerging markets. Using a sample of Chinese-listed companies, we find that the level of green financial constraints is reduced when air quality deteriorates. This effect is more obvious in regions with stronger local government influence or fewer formal environmental regulations. To manage potential self-selection and endogeneity issues, fixed effects (FE), two-stage least squares (2SLS) with instrumental variables (IV), and propensity-score matching (PSM) approaches are used to verify the validity of our results. We link air pollution and financial constraints of green investment, and we fill a literature gap by considering whether the environment can have an impact on corporate green transformation. In the channel analysis, we identify that debt could be an important mechanism through which firms derive fewer green financial constraints. Our findings indicate that air pollution can be a crucial factor restricting corporate green investment and transformation, and managers in the context of emerging markets should be more attentive to green financing.

## 1. Introduction

Although corporations have come to recognize the importance of sustainable development and green activities, lacking adequate financial resources can be a major barrier to firms becoming more environmentally friendly [1]. Under the global green development trend, a green structural change requires long-term commitments [2]; recently, the promotion and far-reaching impact of finance on green development has attracted increasingly more attention [3,4]. However, little research has focused on the financing constraints of green investment at the firm-level. Corporate environmental investments require a large volume of financial resources in the short term [5]. Additionally, these issues may be more significant in an emerging market, where firms find it more difficult to obtain financial resources. When will corporate green investment obtain more financial support or resistance? How does air pollution affect green financial constraints? These are important issues in green development. Therefore, from the perspective of institutional theory, this study first explores the impact and mechanism of external factors, such as air pollution, on green financial constraints.

Institutional theory can better guide this study in its theoretical analysis, especially in the emerging market. Organizational operation is not only affected by economics and technology, but also the whole institutional environment [6]. In the Chinese context, institutional environments are particularly special and important [7]. Chinese governments play an important role in environmental conservation, especially when green GDP has been considered in the KPI of local governments. Both the central government and local governments are under significant pressure to protect the environment. Furthermore, the government can be a “visible hand” to influence corporate operation, since most of the valuable production resources are controlled by various layers of government, especially local governments [8]. Differing from developed countries, government behavior as a special informal institutional factor appears to be more influential in China. Governments that control a large number of vital resources have enough motivation and adequate ability to manage resources to exert influence on corporations’ green transformation under ever-increasing environmental pressures [9]. In this way, air pollution, as an important factor that attracts public attention, will inevitably affect corporate financial constraints of green investment through the influence of governments. We argue that firms located in regions with poor air quality may benefit from lower green financial constraints by securing support from governments.

Based on samples from Chinese listed companies, in this study we investigate the relationship between air pollution and financial constraints of green investment. We find that firms located in regions of poor air quality may experience less green financial constraint, and this relationship is more pronounced in regions where local governments have more influence on the market and formal institutions are weak. Furthermore, government subsidies have been identified as a main channel through which local governments exert their influence.

Our study makes some contributions to the existing literature. First, current studies have identified that air pollution can be harmful to economic development [10], business ethics, management decisions [11,12], and productivity [13]. However, we still know little about how air pollution affects economic development at the level of an individual firm. To the best of our knowledge, this paper is the first to provide empirical evidence on the effect of air pollution on green financial constraints, which is vital for firms’ green transformation.

Second, our paper is also related to the literature on institutional theory. By introducing into the analysis the important informal factor of institutional environments, namely government, this study provides a more specific image of the relationship between air pollution and green financial constraints. Our findings indicate that, in addition to formal regulations that have been identified by previous studies [14], informal institutions can also be a crucial factor for a firm’s green transformation in the context of emerging markets.

Finally, although current studies have identified that corporations play an important role in environmental protection, most of the literature focuses on corporate green investment [15], green innovation [16], and green spending. The financing factor behind these green behaviors has been ignored to a large extent. This study enriches our knowledge by introducing green financial constraints.

## 2. Institutional Background

To a large extent, a country’s economic history can shape its institutions. China followed the economic pattern of the Soviet Union until an economic reform was launched in the early 1980s, from which the Chinese economy transformed into a socialist market economy [17]. In spite of gradually reducing the interference of “visible hands”, Chinese companies are still experiencing the climate of government influence [18]. With fast economic growth during the last three decades, environmental protection has received significant public attention, and Chinese citizens also have gradually come to recognize the importance of environmental quality. Although GDP (Gross Domestic Product) still plays an important role in the KPI (Key Performance Index) of Chinese local governments, environmental problems are receiving increasingly more governmental attention, and governments have come to recognize the importance of corporate environmental investment [19]. As a result, the government has not only issued a series of policies to strengthen administrative supervision but has also tried to help corporations invest in green efforts through various types of support.

In order to reduce finance constraints of environmental investment, the State Council of the PRC (People’s Republic of China) promulgated the “Air Pollution Prevention and Control Action Plan” in 2013. This plan requires banks to provide easier access to finance for green projects. In 2014, as a response to the “12th Five-year Plan for Environmental Protection”, a KPI of green loans was made by the China Banking Regulatory Commission (CBRC), which ensures that commercial banks provide easier access to green projects. At the end of 2015, a guide for green bonds was issued by the National Development and Reform Commission, which encouraged corporations and financial institutions to issue bonds for green projects. As reported by REUTERS, in 2018, Chinese corporations and financial institutions issued USD 30.9 billion in green bonds, which makes China, next to the US, the second largest green bonds market in the world. In June of 2022, the “Green Finance Guidelines for Banking and Insurance” was issued by the CBRC. These guidelines require banks and financial institutions to set KPIs on green financing and rebuild green financing processes to help their customers achieve green transformation. According to the guidelines, the CBRC not only requires banks to provide more loans to environmental protection projects, but also requires specific procedures. In total, by involving financial institutions in providing funds for environmental protection, the possibility of acquisition of green debt financing for corporations has been enhanced.

Based on institutional theory, organizations are influenced heavily by institutional environments, which include both formal factors, such as laws and rules, and informal factors, such as political influence, culture, and public interests [20]. In the developed markets, firms’ operational environments are shaped by formal institutions which are efficient and stable. However, in the emerging markets where there remains a lack of formal institutions, firms have to rely on informal factors [21]. Particularly, in the context of China, governments have been identified as one of the most important stakeholders and provide not only resources but also legitimacy, which is crucial to the survival of organizations in emerging markets and the firms. Therefore, Chinese governments fill the void that has been left by the lack of formal market institutions and, as a result, governments have more opportunities to influence firms.

## 3. Literature Review

The economic consequences of ambient air pollution have been the subject of extensive research. Previous research has attempted to understand the impact of air pollution through various lenses, including macroeconomics [22], labor economics [23], and behavioral finance [12]. The existing literature indicates that ambient air pollution can have serious negative impacts on working durations [23] and worker productivity [13]. However, little attention has been paid to consequences at the firm level. Until recently, a small but growing amount of literature has paid attention to this important topic.

Recently, more and more studies have come to recognize that air pollution not only has impacts on macroeconomic development and human health, but also affects corporation performance and policy. Mood has been identified as a main mechanism through which air pollution affects firms. Based on site visiting data of Chinese analysts, Dong et al. [24] found that air pollution can make analysts more pessimistic, and, thus, those analysts visiting higher pollution cities are likely to issue more conservative earning forecasts. Similarly, based on data from the top managers of Chinese listed companies, Hu et al. [25] found that managers in high-pollution areas were more likely to make poor decisions and this led to lower financial reporting quality; this finding is attributed to the emotional damage that air pollution has on people. In addition, the negative impact on mood, current studies have also found that air pollution can negatively affect firms through brain drain. Firstly, because air pollution causes serious harm to human health, high-skill people are likely to move to more environmentally friendly regions, which reduces the competitiveness of companies in high-pollution areas [26,27]. Secondly, for the same reason, the firms located in poor air quality cities have to pay more for retaining top managers [28]. Therefore, most current studies argue that air pollution has a negative impact on firms. Although the literature has indicated that air pollution may increase the risk of firms, little attention has been paid to its effect on financial constraints, especially green financial constraints. Based on institutional theory, this study tries to explore this knowledge by linking air pollution and green financial constraints.

## 4. Hypotheses Development

### 4.1. Air Pollution and Financial Constraints of Green Investment

In this study, we shed new light on the relationship between air pollution and the financial constraints of green investment. Although numerous studies have argued for the importance of environment protection and corporate green transformation [29,30], financial constraints can be a critical factor affecting firms’ green investments. Because they are different from common investments, green investments are more likely to rely on firms’ own cashflows and, thus, suffer from more serious financial constraints. Based on the institutional environment of China, we argue that the firms located in high air pollution areas, suffering from poor air quality, face lower green financial constraints.

Governments have enough motivations to remove barriers for firms’ green financing. Based on city-level panel data in China, Hao et al. [10] showed that environmental problems, such as air pollution, can have a harmful effect on economic development. The public sector is paying more and more attention to the issue of air pollution, which, in turn, forces governments to engage more deeply in the protection of the environment. Governments in more air polluted regions may be under greater public pressure and they, therefore, need to engage more deeply in creating better conditions for green investments. Since financial constraint is a major obstacle to green investment, linking external finance (e.g., banks) and green projects and reducing the information asymmetry between them should be a strategy for governments.

Governments are not only motivated to reduce green financial constraints, but they also have the power to do this. Information asymmetry has been identified as a main mechanism through which financial constraints arise [31,32]. Green investments are often involved in polluted projects, and to identify or assess the environmental risk requires professional knowledge and inside information. Nevertheless, banks and other external financial institutions lack that information and the access to that information [33], which generates substantial information asymmetry. Chinese governments could play an important role in reducing information asymmetry between the firms with green investments and external investors. In the context of China, certification or endorsement from governments may reduce the information asymmetry substantially and is quite significant in the acquisition of external finance. In opposition to other external organizations, which cannot gain enough inside information into corporations, governments in China are more likely to be involved in a firm’s operation and could have more information on whether the firms qualify for finance. Li et al. [34] identified that high-risk Chinese innovational firms have easier access to external finance by obtaining government endorsement, because this recognition suggests a solid message to the capital market that these firms are high quality. Therefore, external investors could gain more inside information through the government.

Therefore, under increasing public environmental pressures, governments may relieve a firm’s green financial constraints by reducing information asymmetry between external capital providers and firms.

**Hypothesis** **1.** *Because of the support from governments, firms suffering from poor air pollution have lower financial constraints in green investments.*

### 4.2. The Moderating Effect of Government Influence

Institutions are defined as a combination of various economic, social, cultural, and political regulations, which organizations are required to comply with in order to gain support and legitimacy [35]. Informal institutions are treated as significant factors for firms, especially in emerging markets [36,37]. In regions with weak institutional environments, informal methods, such as administrative policy as a substitution for formal institutions, can have more influence on firms.

If air pollution impacts financial constraints of green investments, our next objective is to identify the boundary conditions of the effects of air pollution. As discussed above, air pollution affects financial constraints through the engagement of governments; therefore, it is reasonable to propose that the power and influence of governments could be an important contingent factor affecting this relationship. Although an “Open Policy” has been taking place in China for around 40 years, the degree of the influence of governments varies across different regions, since the reforms have been introduced gradually [38]. Apart from having a greater impact on key resource collection [39], a more powerful government could also lead to greater environmental pressure. In those regions where local governments have more influence, authorities can also gain more attention from the public and media. Hence, people tend to attribute air pollution to the lax enforcement of local environmental protection bureaus, which will result in a greater environmental pressure on local governments.

Consequently, we propose the following:

**Hypothesis** **2a.** *The negative relationship between air pollution and financial constraints of green investments remains in the regions with stronger government influence.*

**Hypothesis** **2b.** *The negative relationship between air pollution and financial constraints of green investments does not exist in the regions with weaker government influence.*

Following the logic as we discussed earlier, if the negative relationship between air pollution and financial constraints of green investments could be moderated by government influence, as another side of the same coin, the impact of formal institutions, such as regulations and laws, should also exist. In the last decade, various levels of governments and the People’s Congress responsible for passing laws in China have issued many regulations and laws surrounding environmental protection. Up to 2017, 798 local regulations have been issued by various levels of regional governments. The corporations in regions with more environment regulations must follow these regulatory guidelines rather than administrative policy; as a result, governments have less room to influence firms. Furthermore, in regions where environmental regulations are well established, other types of regulations, such as regulations in finance, may also be more sophisticated, which could limit the influence of governments. Hence, we propose that environmental legislations, such as a formal institution guiding firms’ green strategies, could reduce the motivation and ability of governments to interfere and, as a result, they may have a moderating effect on the relationship between air pollution and financial constraints of green investments.

**Hypothesis** **3a.** *The negative relationship between air pollution and financial constraints of green investments remains in regions with fewer formal environmental regulations.*

**Hypothesis** **3b.** *The negative relationship between air pollution and financial constraints of green investments does not exist in regions with more formal environmental regulations.*

## 5. Methods

### 5.1. Sample Selection and Data Sources

The sample of this study consists of Chinese A-share firms listed on the Shanghai Stock Exchange and Shenzhen Stock Exchange from 2004 to 2016. The raw data were processed as per the following rules: (1) we eliminated companies with no environmental investment activities and missing information, (2) companies were listed in the current year, (3) financial firms were excluded from the sample, (4) the continuous variables were winsorized at the 1% and 99% quantiles by year. As a result, our final sample comprised 653 companies with an unbalanced panel, in 18 sectors (mining, pharmaceuticals, etc.), for a total of 2248 observations.

The financial and governance data were obtained from the China Stock Market and Accounting Research (CSMAR) Database. CSMAR is an economic and financial database developed in conjunction with China’s current conditions. It is mainly based on the needs of academic research, drawing on the professional standards of the internationally renowned databases such as CRSP, Compustat, I/B/E/S, Thomson, etc., which are widely used in China’s relevant economic research [40,41]. For example, referring to the existing literature, the data of the dependent variable corporate environmental protection investment in the model come from the corporate financial statement note data in CSMAR. The data of the independent variable air quality come from the Social Economic Data and Applications Center (SEDAC) hosted by Columbia University. The data for the government influence and formal environmental regulations come from the Marketization Index Report sponsored by the National Economic Research Institute (NERI) and the China Environment Yearbook sponsored by the Ministry of Ecology and Environment of the PRC. These are described in detail in the variables section.

### 5.2. Variables

#### 5.2.1. Financial Constraints of Green Investments

For the measurement of corporate financial constraints, following Fazzari et al. [42], investment cash flow sensitivity (ICFS) is frequently adopted. In the ideal scenario, corporations could obtain financial resources at any time when they have an investment opportunity. However, expensive external financing costs due to financing frictions and information asymmetry may prevent firms from investing in some projects or force firms to use their internal funds to invest. Under such conditions, the investment expenditures would be highly correlated with the net amount of cash flow, which means higher ICFS. Drawing on this idea, we use environmental investment cash flow sensitivity (EICFS) as the proxy of financial constraints of environmental investments.

For the environmental investments, referring to the existing research, such as Li and Lu [43], we measure the dependent variable Environmental Protection Investment (EPI) by calculating the ratio of a firm’s current year’s environmental capital expenditures and the initial fixed asset. The environmental capital expenditure is a direct measure of environmental actions taken by companies, as suggested by Patten [44]. We obtain this from the annual financial statements of the enterprise, which reflect the increase in environmental investment in the current period.

#### 5.2.2. Air Pollution

In China, particulate matter of 2.5 μm (PM2.5) is one of the most concerning types of air pollutants [45]. We estimate the annual average concentration of PM2.5 in cities across China, by using ArcGIS software to resolve the global annual PM2.5 concentrations grid dataset published by the Social Economic Data and Applications Center (SEDAC) [46]. The SEDAC is a data center in NASA’s Earth Observing System Data and Information System, hosted by CIESIN at Columbia University. This global PM2.5 grids dataset combines AOD retrievals from multiple satellite instruments including the NASA Moderate Resolution Imaging Spectroradiometer (MODIS), Multi-angle Imaging Spectro Radiometer (MISR), and the Sea-Viewing Wide Field-of-View Sensor (SeaWiFS). These data are widely used in environment-related research fields [47], as well as in China’s environmental research [48,49]. Furthermore, some studies suggest that AOD is an objective measure immune from manipulation [50]. Therefore, PM2.5 concentrations can be employed as an effective indicator to measure air pollution.

#### 5.2.3. Government Influence and Formal Environmental Regulations

Considering the existing literature, first, to measure the power and influence of local governments, we use the indices of marketization of China’s Provinces provided by the National Economic Research Institute (NERI), which reflects the degree of government intervention in the market and fairness in economic development. The NERI index captures the following aspects of regional development: relationship between government and markets; development of non-state sectors; development of product markets; development of factor markets; development of market intermediaries; and the legal environment. This dataset has been routinely applied in Chinese management and economics research [51,52] and is continuously updated with data up to 2016 [53]; a higher index value represents more developed market institutions [54]. Specifically, this means that the larger the index value, the lower the degree of government intervention.

In addition, according to China’s regional development differences, the eastern region is relatively more developed and the marketization level is higher [14], while the economic development level of the central and western regions is lower. Therefore, we also measure the power of governments according to whether the company is from the eastern region according to the regional division of the National Bureau of Statistics.

Then, to measure the degree of formal environmental regulations, we use the number of environmental policies and regulations newly implemented every year in each region. Under the Chinese legislative system, the regional People’s Congresses and their standing committees are authorized to formulate and promulgate local laws. Therefore, the number of local laws and regulations varies across different regions. These data come from the China Environmental Yearbook, which is sponsored by the Ministry of Ecology and Environment of the PRC. The editorial committee of the yearbook is composed of officials from the Ministry of Ecology and Environment of the PRC and the Department of Ecology and Environment in each province.

### 5.3. Model

According to the investment cash flow sensitivity idea proposed by Fazzari et al. [42], we examine the effect of air pollution on EICFS by estimating the following multiple regression model:

(1) $$\begin{array}{l} EPI_{i,t}=\beta_{0}+\beta_{1}CF_{i,t}+\beta_{2}CF_{i,t}\times AIR_{i,t}+\beta_{3}AIR_{i,t}+\beta_{4}Size_{i,t}+\beta_{5}Lev_{i,t}\\ \;+\beta_{6}Cash_{i,t}+\beta_{7}Q_{i,t}+\beta_{8}Growth_{i,t}+\beta_{9}PPE_{i,t}+\beta_{10}First_{i,t}\\ \;+\beta_{11}Dual_{i,t}+\beta_{12}State_{i,t}+\beta_{13}Age_{i,t}+\beta_{14}GDP_{i,t}\\ \;+\beta_{15}Market_{i,t}+\delta_{k}+\omega_{t}+\varepsilon \end{array}$$

where $\beta_{1}$ reflects the dependence of EPI on internal cash flow; $\beta_{2}$ reflects the impact of Air on EPI financing dependence;$\;\delta_{k}$ and $\omega_{t}$ are industry and year fixed effects, respectively; ε is the regression error term. According to the theoretical analysis above, we would expect a positive sign on $\beta_{1}$ and a negative sign on $\beta_{2}$, the coefficient for cash flow (CF) and the multiplication (CF ×AIR).

We follow prior studies and control a set of firm and regional variables [8,43,55]: Size (the natural logarithm of firm’s total assets); Lev (the ratio of total liabilities to total assets); Cash (the ratio of cash balance to total assets); Q (Tobin’Q); Growth (the annual sales growth rate); PPE (the ratio of fixed assets to total assets); First (the percentage of shares held by the largest shareholder); Dual (a dummy variable, 1 whether the chairman and CEO are the same person); State (a dummy variable, 1 for firms whose controlling shareholder is the state and 0 otherwise); Age (the number of years the firm has been publicly listed); GDP (The natural logarithm of regional GDP); and Market (regional marketization index). Since firms are more likely to be green to obtain social legitimacy and reduce business risks [56,57], we expect the coefficients of Size, PPE, State, Age, and GDP to be negative, while the coefficients of Q, First, Growth, and Market should be positive. Since corporate environmental investment needs a lot of financial resources [58,59], we expect the coefficients on Lev, Cash and Dual to be positive. The details of variables included in this study can be seen in Table 1.

Given that the dependent variable has been censored on the left side, we use censored regression (Tobit) to retest the results of our proposed model with standard errors clustering at the firm level.

## 6. Results

In this section, we assess whether the evidence shows that firms in poor air quality regions enjoy a lower green financial constraint, and whether this relationship is more pronounced in regions where local governments have more influence on the market or in those with fewer formal environmental institutions. Local governments help firms to be green through two main channels: bank loan and government subsidies.

### 6.1. Descriptive Statistics of Variables

In an attempt to provide an overview of corporate environmental protection investment and its influence, in Table 2 we report descriptive statistics of the main variables used in our research. This is winsorized at the 1st and 99th percentiles for all continuous variables used in the model. As shown in Table 2, the distribution of EPI is biased; its median is much smaller than its mean. This indicates that only some Chinese listed companies are willing to conduct environmental investment.

With reference to the “Guidelines for Industry Classification of Listed Companies” (2012 version) published by the China Securities Regulatory Commission, the sample companies in this study include 15 industry categories, such as agriculture, mining, manufacturing, and utilities, covering 80% of all industry categories. Due to the large number and high proportion of manufacturing companies in China’s capital market, this study further subdivided the manufacturing industry and consolidated some service companies with similar business models, as shown in Table 3. Table 3 presents the description of the EPI of companies in various industries. We find that there are significant differences in EPI between different industries in China. In addition to specialized environmental governance industries, the average EPI of companies in construction, real estate, and petrochemical manufacturing industries is relatively high, while the average EPI of companies in transportation, agriculture, and food manufacturing is relatively low. Therefore, regression analysis based on our sample companies can objectively and comprehensively reflect the population.

Table 4 displays pairwise Spearman rank-order correlations. EPI and CF are significantly correlated (correlation coefficient = 0.124), primarily showing that corporate green investment is affected by its net operating cash flow. The assets of corporation, cash holding amount, market value, and the nature of property can also affect corporate green investment.

### 6.2. Regression Analysis

Table 5 reports the analysis of the impact of air pollution on EICFS. In agreement with Hypothesis 1, we find a significant, negative relationship between interaction (AIR × CF) and EPI (b = −0.142, *p* < 0.01). This hypothesis predicted that firms in areas with poor air pollution would have lower EICFS. In Column (1) and Column (2), we present the result of model (1) without controlling variables. In Column (3) and Column (4), we estimate the impact of air pollution on green financial constraint after including control variables. Obviously, CF is positively and significantly correlated with EPI (*p* < 0.001), and the coefficients of the interaction term (AIR × CF) are significantly negative. Hence, H1 is supported.

In order to test whether the relationship between air pollution and green financial constraint is influenced by government influence, we conduct two group tests in Table 6 and Table 7 separately. In Table 6, samples are grouped by the degree of government intervention, according to whether the degree of marketization in the company’s region is higher than the median. Column (1) and Column (2) separately present the analysis of EICFS and its relationship with air pollution in the regions with low levels of government influence, and the analysis in the regions with high levels of government influence is presented in Column (3) and (4). The results show that EICFS exists in both high and low marketization regions. While in regions of high marketization air pollution has no significant effect on EICFS, in low marketization regions there is a significantly negative relationship between air pollution and EICFS. The difference between these two regions is significant (chi2 = 4.89, *p* = 0.027). This illustrates that government intervention plays a key role in the relationship between corporate environmental investment and air pollution, supporting Hypothesis 2b.

Similarly, in Table 7, samples are grouped by the degree of government influence, according to whether the company is in the eastern region. The east of China is more developed, with a high level of marketization. Governments have a stronger ability to intervene in economic resources in inland regions, which may have a more significant moderating effect on the impact of air pollution on green financial constraints. The results show that EICFS exists in both eastern and non-eastern regions (Table 7). There is a non-significant relationship between air pollution and EICFS in the eastern region in Column (2), and air pollution is significantly negatively correlated with EICFS in the non-eastern region in Column (4). This shows that government intervention affects the impact of air pollution on EICFS. The difference between these two regions was significant (chi2 = 3.21, prob > chi2 = 0.073).

To test whether the effect of air pollution on financial constraints of green investments varies with formal environmental regulations, we partitioned the sample at the median values of the degree of local environmental legislation where the company is located. Then, we, respectively, estimated the Tobit model. In Table 8, we find that the coefficient on CF is positive and significant at 1% in all columns, consistent with previous results. At the same time, we find that the coefficient on AIR × CF is negative and significant at 1% for the subsample companies located in regions where the formal environmental regulations are less (Column (2)), but it is not different from zero in the subsample companies located in regions where the formal environmental regulations are strong (Column (4)). Furthermore, we find that the difference in the coefficient on AIR × CF is statistically significant in this comparison, χ^2^ = 4.91, ρ = 0.026. Tests of coefficient difference imply that the effect of air pollution on green financing constraints would strengthen significantly if the company is located in regions with fewer formal environmental regulations.

### 6.3. Robust Tests

In order to verify the robustness of our baseline estimations illustrated in Table 5, we re-estimated model (1). Due to the potential endogeneity, which may bias our results, we used several approaches to mitigate endogeneity concerns and conduct robustness tests.

First, concern arises that the result could be driven by firm characteristics that affect firms’ green investment choices. Thus, we introduce a firm fixed-effects model that controls for time-invariant unobserved firm characteristics. We use the ordinary least squares multiple regression model (OLS) and fixed effect model (FE) to retest the impact of air pollution on the EICFS. The results (Table 9) confirm the negative and significant effects of air pollution on EICFS, indicating that the conclusions of this article are robust.

Second, a concern with our main regressions is that the air pollution may not be exogenous, and some unobserved variables could link the air pollution and green financial constraint. To address this concern, we use the instrumental variable model (IV) based on the two-stage least squares method (2SLS). We seek instruments that proxy for air pollution that are not related to firm characteristics. According to previous findings [46,60], meteorological conditions (wind stress, rain amount, relative humidity) have a clear effect on the concentration of PM2.5. For particulate matter, high humidity corresponds to low PM2.5 concentrations, and there is a negative relationship between precipitation and PM2.5 concentrations. Thus, we use the relative humidity and precipitation as our instrumental variable to capture the “exogenous” part of local air pollution.

We regress our integrity variable on the two instruments and control variables from the baseline model and find that the *p* values of Anderson LM statistic and Sargan statistic are all less than 0.05, rejecting the null hypothesis that each endogenous variable is insufficiently identified or over identified. The Cragg–Donald Wald F statistic is 6.906, rejecting the null hypothesis that each endogenous variable is weakly identified. We retain the predicted values of these integrity variables in our second regression. Not surprisingly, the coefficient on the interaction is significantly negative, consistent with the previous results. We then perform Hansen’s (1982) over-identification test, showing that all *p* values are more than 0.1, suggesting that our instrumental variables are suitable and exogenous. Overall, these results (Table 10) suggest that our findings are robust.

Third, another concern is that it may be possible that our results, so far, suffer from a self-selection bias. That is, the location of companies in areas with severe air pollution may be the result of government influence and, thus, not random. Generally, there are significant differences in the characteristics of industrial enterprises in regions with different levels of air quality in China, which may affect the main conclusions. For example, Hebei is a representative province with poor air quality and a traditional heavy industry base; companies in Hebei are usually large, state-owned, and heavy industrial enterprises. These companies are more likely to obtain government financial supports and subsidies. Therefore, we utilize the propensity score matching (PSM) [61] to alleviate the selection bias concern, through a one-to-one matching with firm characteristics, including Size, Lev, PPE, Dual, and State. After matching, the total sample size of the two groups is 1438. Based on this PSM sample, the results are shown in Column (1) of Table 11 and are consistent with the previous results.

Furthermore, to capture the air pollution more fully, we consider using another indicator to capture this dependent variable. Referring to existing studies [62], Air Quality Index (AQI) values, as another indicator, are publicly available from the State Environmental Protection Agency (SEPA, www.mee.gov.cn). AQI values are a scientific measure of air quality designed to inform the public about air pollution and the potential impacts on human health. It concentrates on the pollutants PM10, NO2, SO2, CO, and O3, which are monitored and converted to AQI each day. A higher AQI value indicates more serious pollution and higher risks to human. We re-estimate our baseline model using the natural logarithm of the AQI annual average for each city. Since the national AQI was published in 2014, the sample data were reduced to 673. The results are reported in Table 11. All our results continue to hold (see Column (2) in Table 11).

Last, in order to test the potential competitive hypothesis that the negative relationship between air pollution and green financial constraint is not caused by local government being stimulated, but by less green investment of enterprises in places with poor air quality, we conduct univariate testing of EPI of enterprises in regions with different air quality. Table 12 provides the comparison of EPI with two-sample t-test for differences across the two sum-samples. The EPI of enterprises in regions with poor air quality is not significantly more than those in regions with good air quality (*p* > 0.1). There are no significant differences between the two samples. Thus, in regions with poor air quality, the reduction in green financial constraint does not result from lower green investment but is caused by government attention and support.

### 6.4. Further Analysis

In Section 2, we argue that air pollution results in lower financial constraints by government support, which, in turn, results in lower EICFS. We examine this main effect and perform group tests by introducing a moderating variable. In this section, we explicitly examine these proposed channels by employing the three-step mediation analysis suggested by Baron and Kenny [63]. Step 1 is to estimate the proposed model without the mediating variable, which is what we report in Table 5, Model 4. Recall that the coefficient of interest, AIR × CF, is negative and significant (−0.232, *p* < 0.01). This is the effect to be mediated.

Step 2 is to show that the independent variable of interest, AIR, does in fact lead to the proposed changes in the mediating variables, that is, to higher debt financing (DF). In China, the government has begun to push commercial banks to develop green finance businesses and provide more green loans and green bonds. As discussed in Section 2, by involving financial institutions to provide funds for environmental protection, the possibility of acquisition of green debt financing for corporations has been enhanced. Therefore, we use debt financing as a moderator. DF is defined as the net debt financing for the current period by initial fixed assets. We estimate the following models:

(2) $$\begin{array}{l} DF_{i,t}=\beta_{0}+\beta_{1}AIR_{i,t}+\beta_{2}Size_{i,t}+\beta_{3}Lev_{i,t}+\beta_{4}Cash_{i,t}+\beta_{7}Q_{i,t}+\beta_{5}Growth_{i,t}\\ \;+\beta_{6}PPE_{i,t}+\beta_{7}First_{i,t}+\beta_{8}Dual_{i,t}+\beta_{9}State_{i,t}+\beta_{10}Age_{i,t}\\ \;+\beta_{11}GDP_{i,t}+\beta_{12}Market_{i,t}+\delta_{k}+\omega_{t}+\varepsilon \end{array}$$

Finally, in step 3, we estimate the full model including our proposed mediation variables, the interaction term AIR×DF. The results are presented in Table 13. Consistent with our expectation, a higher AIR is positively associated with future debt financing in model 1 (0.837, *p* < 0.05). Model 2 repeats the results from Table 5, Model 4 (i.e., the effect to be mediated). Importantly, the magnitude of the coefficient on AIR × CF exhibits a statistically significant (*p* < 0.01) rise from −0.232 to −0.063 (*p* < 0.05, model 3) after including the mediating variable AIR × DF. That is, the negative relationship between AIR and EICFS has weakened. We conclude that, consistent with our theory, our finding of a negative association between AIR and EICFS is at least partially attributable to an increase in debt financing.

Thus, in this section, according to the investment cash flow sensitivity idea, we found that air pollution is negatively correlated with EICFS, that is, firms in regions with poor air pollution have lower financial constraints of green investments (Section 6.2). This result is robust (Section 6.3). Then, we conducted group tests and found that the negative relationship between air pollution and green financial constraints was significant in the regions with high level of government influence or fewer environmental regulations (Section 6.2). To some extent, these differences in comparison demonstrate the negative correlation between air pollution and corporate financing constraints, which may be caused by the government’s support for green financing when the government is under higher environmental protection pressure. Thus, we further conducted a three-step test with debt financing as a moderator, and the results support our hypothesis more directly (Section 6.4).

## 7. Discussion

### 7.1. Theoretical and Practical Implications

To the best of our knowledge, this paper is the first to link air pollution and green financial constraints. By drawing on institutional theory, this study explored the mechanisms affecting the relationship between air pollution and green financial constraints. As there is a trend of paying more attention to the “visible hands” of government, our findings could help us better understand how governments engage in the green financial market and help firms become greener.

This study has several practical implications for policy makers. Our findings indicate that financing constraints could be a major reason why firms spend less on green projects. In the context of China, governments play an important role in corporate green investments, and can help high-polluting firms become greener by offering easier access to financing. Therefore, a high-efficiency green capital market is crucial for reducing corporate green financial constraints. Second, our findings show that, as well as the negative effects on public health, air pollution can also impact corporation green performance. Hence, when governments make an attempt to mitigate air pollution, alongside individual welfare, corporation performance and economics growth can also benefit.

### 7.2. Limitations and Future Research

Our findings have effectively supplemented and expanded the current research in the field of environmental economics at the micro level, but we also recognize some limitations to this study. First, in the informal factors, we mainly focused on government intervention, without considering the participation of non-profit organizations, media attention, and others. Thus, green financial constraint could be affected by various factors, including external and internal factors; however, in this paper, we only focused on the external factors. Lastly, we used a database from the Social Economic Data and Applications Center to measure the level of air pollution. Although this database has been widely used in the field of environmental research, it has not been updated since 2016. Therefore, we cannot test our hypotheses based on more recent data. 

In view of these limitations, we have some suggestions for future research. First, the research could be expanded from the perspective of other informal factors, focusing on the role of the media, the public, and some organizations. Second, besides the institutional environment, internal factors can also impact firms’ green transformation. Hence, it would make sense to upgrade the research by drawing on the perspective of corporate governance. Finally, a database containing up to date evidence could be useful. Additional data on air pollution could be collected via data-mining from other sources for future research.

## 8. Conclusions

Although the consequences of air pollution have been widely documented by researchers, most current research is based on the effect of medical and psychological factors, and it focuses on either the macro level or the individual level; little attention has been paid to the impact on corporate financial constraints, especially under a specific institutional context of emerging markets. The objective of this study was to investigate whether air pollution may attract attention from governments and push governments toward green investments, in turn helping firms obtain access to financing, which may finally reduce the firms’ green financial constraints.

Based on the samples of China’s listed firms, we found that the firms located in the regions with higher air pollution may face lower green financial constraints, and we attribute this phenomenon to the institutional environment. In line with the logic mentioned above, we further studied whether the impact of air pollution could vary across different regions, since regions differ greatly in China. We found that the negative relationship between air pollution and green financial constraints was significant in the regions with a high level of government influence or fewer environmental regulations, while it was not significant in the regions where governments did not play a dominant role or where environmental legislation was not well established. Finally, upon analyzing mechanisms, we found that bank debt plays an important mediating role.

## Figures and Tables

**Table 1:** Variable definitions.

Variable	Name	Measurement
**Dependent Variable**		
Environmental Investment	EPI	Environmental capital expenditure/initial fixed assets
**Independent Variables**		
Net cash flow	CF	Operating cash flow/initial fixed assets
Air pollution	AIR	PM2.5 concentrations in each city
**Control Variables**		
Size	Size	The natural logarithm of the total assets
Leverage	Lev	The ratio of total liabilities to total assets
Cash	Cash	The ratio of cash balance to total assets
Tobin’s Q ratio	Q	(Market value of equity + book value of liability)/total assets
Growth	Growth	Operating income growth/previous operating income
Fixed assets ratio	PPE	The ratio of fixed assets over total assets
Majority shareholder	First	Percentage of shares held by the largest shareholder
CEO Duality	Dual	If the chairman and CEO are the same person, 1, otherwise 0
State	State	If the firm belongs to state-owned firm, State = 1, 0 otherwise
Age	Age	Years elapsed since the firm was listed
Economic growth	GDP	The natural logarithm of regional GDP
Government influence-market	GI-market	The marketization index
Government influence-region	GI-Region	If the register place of the firm locates in the eastern region, GI_Region = 1, 0 otherwise
Formal environmental regulations	Elaw	The number of the newly formulated environmental regulations in each province
Net debt financing	DF	Net debt financing/initial fixed assets
Industry	Indus	Industry dummy variables
Year	Year	Year dummy variables

**Table 2:** Descriptive statistics.

Variable	N	Mean	Std. Dev.	Median	Min	Max
EPI	2248	0.045	0.158	0.004	0	1.780
CF	2248	0.229	0.657	0.174	−5.020	8.870
AIR	2248	0.412	0.163	0.401	0.026	0.926
Size	2248	21.700	1.370	21.600	18.400	25.700
Lev	2248	0.500	0.180	0.515	0.048	0.928
Cash	2248	0.147	0.102	0.119	0.004	0.616
Q	2248	2.100	1.370	1.690	0.795	15.10
Growth	2248	0.268	1.790	0.060	−0.928	49.200
PPE	2248	0.382	0.238	0.354	0.008	3.330
First	2248	0.382	0.159	0.364	0.051	0.840
Dual	2248	0.145	0.352	0	0	1
State	2248	0.688	0.463	1	0	1
Age	2248	10.600	5.360	11	1	23
GDP	2248	9.810	0.845	9.860	6.820	11.300
GI-market	2248	7.300	1.870	7.160	2.330	11.700
GI-region	2248	0.502	0.500	1	0	1
Elaw	2248	2.190	2.780	1	0	23
DF	2248	1.640	4.420	0.817	0	101.000

**Table 3:** Descriptive statistics of industry characteristics of the enterprises’ EPI.

Sector	N	Percent	Mean	Median	Min	Max
Agriculture	60	2.67%	0.012	0.002	0	0.142
Mining and quarrying	100	4.45%	0.030	0.004	0	1.210
Manufacture of foods	196	8.72%	0.007	0.003	0	0.073
Manufacture of textiles, clothing, and related products	84	3.74%	0.044	0.005	0	1.270
Manufacture of paper and related products	98	4.36%	0.034	0.004	0	1.530
Manufacture of petroleum and chemical raw material production	373	16.59%	0.053	0.006	0	1.780
Pharmaceuticals	175	7.78%	0.032	0.004	0	1.530
Manufacture of chemicals (fiber, rubber, plastic, etc.)	149	6.63%	0.039	0.006	0	0.991
Smelting and processing of metal	253	11.25%	0.026	0.007	0	0.852
Manufacture of machinery (general/special purpose)	157	6.98%	0.041	0.006	0	1.090
Manufacture of equipment (electrical and electronic equipment) communication equipment manufacturing	146	6.49%	0.070	0.004	0	1.780
Utilities (electric power, heat power, gas, tap water)	193	8.59%	0.040	0.003	0	0.991
Construction	37	1.65%	0.110	0.013	0	0.978
Wholesale and retail trades	40	1.78%	0.052	0.003	0	0.991
Transport, storage, and postal services	36	1.60%	0.003	0.001	0	0.067
Real estate	36	1.56%	0.088	0.032	0	0.682
Administration of water, environment, and public facilities	43	1.60%	0.126	0.072	0	1.110
Other services	72	2.72%	0.066	0.002	0	1.310
Total	2248	100.00	0.045	0.004	0	1.780

**Table 4:** Bivariate correlation analysis of main variables.

Variables	EPI	CF	AIR	Size	Lev	Cash	Q	Growth
EPI	1.000							
CF	0.124 *	1.000						
AIR	−0.003	0.013	1.000					
Size	−0.110 *	0.080 *	0.034	1.000				
Lev	−0.035	−0.055 *	0.034	0.403 *	1.000			
Cash	0.141 *	0.077 *	0.012	−0.078 *	−0.305 *	1.000		
Q	0.086 *	0.069 *	−0.049	−0.214 *	−0.334 *	0.179 *	1.000	
Growth	0.021	−0.023	0.016	−0.091 *	0.029	0.030	0.039	1.000
PPE	−0.090 *	−0.066 *	−0.024	0.097 *	0.044	−0.330 *	−0.082 *	−0.139 *
First	−0.012	0.023	0.058 *	0.300 *	0.033	−0.003	−0.067 *	−0.022
Dual	0.061 *	0.008	−0.012	−0.102 *	−0.086 *	0.060 *	0.075 *	−0.029
State	−0.084 *	−0.040	0.054	0.206 *	0.231 *	−0.154 *	−0.174 *	0.019
Age	−0.076 *	−0.038	−0.025	0.228 *	0.240 *	−0.206 *	−0.067 *	0.001
GDP	−0.009	0.017	0.306 *	0.130 *	−0.090 *	0.082 *	0.061 *	−0.059 *
Market	−0.000	0.017	0.364 *	−0.019	−0.111 *	0.100 *	−0.056 *	−0.008
	PPE	First	Dual	State	Age	GDP	Market	
PPE	1.000							
First	0.148 *	1.000						
Dual	−0.020	−0.126 *	1.000					
State	0.191 *	0.240 *	−0.189 *	1.000				
Age	−0.017	−0.121 *	−0.080 *	0.211 *	1.000			
GDP	−0.137 *	−0.135 *	0.101 *	−0.222 *	0.097 *	1.000		
Market	−0.154 *	−0.071 *	0.073 *	−0.184 *	−0.043	0.604 *	1.000	

Notes: Pearson correlation coefficients; * *p* < 0.01.

**Table 5:** The impact of air pollution on green financial constraint.

Variables	(1)	(2)	(3)	(4)
CF	0.074 ***	0.080 ***	0.075 ***	0.081 ***
	(14.59)	(15.60)	(14.68)	(15.76)
AIR × CF		−0.227 ***		−0.232 ***
		(−6.53)		(−6.80)
AIR		0.003		0.027
		(0.13)		(1.18)
Size			−0.016 ***	−0.017 ***
			(−4.81)	(−5.08)
Lev			0.082 ***	0.081 ***
			(3.52)	(3.53)
Cash			0.185 ***	0.186 ***
			(4.90)	(4.98)
Q			0.002	0.002
			(0.50)	(0.52)
Growth			0.000	0.001
			(0.13)	(0.37)
PPE			−0.023	−0.020
			(−1.36)	(−1.19)
First			0.018	0.014
			(0.75)	(0.59)
Dual			0.017 *	0.017 *
			(1.80)	(1.76)
State			−0.008	−0.010
			(−0.99)	(−1.19)
Age			−0.001	−0.001
			(−0.84)	(−0.79)
GDP			−0.016 **	−0.017 **
			(−2.28)	(−2.44)
Market			0.004	0.003
			(1.34)	(1.00)
Cons	−0.037	−0.040	0.341 ***	0.362 ***
	(−1.31)	(−1.41)	(4.21)	(4.48)
Sigma_cons	0.154 ***	0.153 ***	0.152 ***	0.150 ***
	(63.99)	(64.00)	(63.93)	(63.94)
Industry fixed effect	yes	yes	yes	yes
Year fixed effect	yes	yes	yes	yes
Observations	2248	2248	2248	2248
χ^2^-statistic	318.300	360.621	392.904	440.431

Note: * *p* < 0.10, ** *p* < 0.05, *** *p* < 0.01; t-values are in parentheses.

**Table 6:** Grouping based on government influence- marketization.

Variables	High Marketization Region (Low Government Influence)		Low Marketization Region (High Government Influence)	
	(1)	(2)	(3)	(4)
CF	0.127 ***	0.129 ***	0.034 ***	0.052 ***
	(16.25)	(15.59)	(5.40)	(7.08)
AIR × CF		0.047		−0.216 ***
		(0.79)		(−4.61)
AIR		−0.039		0.023
		(−1.09)		(0.72)
Size	−0.023 ***	−0.023 ***	−0.007 *	−0.007 *
	(−4.80)	(−4.82)	(−1.76)	(−1.68)
Lev	0.116 ***	0.117 ***	0.035	0.032
	(3.51)	(3.55)	(1.15)	(1.06)
Cash	0.131 **	0.133 **	0.151 ***	0.161 ***
	(2.38)	(2.43)	(3.10)	(3.31)
Q	−0.001	−0.001	−0.001	0.000
	(−0.16)	(−0.18)	(−0.21)	(0.02)
Growth	0.001	0.001	−0.016 *	−0.015 *
	(0.62)	(0.62)	(−1.93)	(−1.91)
PPE	0.003	0.003	−0.020	−0.014
	(0.17)	(0.17)	(−0.74)	(−0.51)
First	−0.008	−0.007	0.041	0.037
	(−0.26)	(−0.22)	(1.25)	(1.12)
Dual	0.028 **	0.027 **	0.003	0.001
	(2.00)	(1.98)	(0.21)	(0.10)
State	−0.013	−0.012	−0.005	−0.005
	(−1.10)	(−1.06)	(−0.48)	(−0.45)
Age	−0.000	−0.000	−0.002	−0.001
	(−0.11)	(−0.04)	(−1.49)	(−1.46)
GDP	−0.008	−0.002	−0.015*	−0.012
	(−0.66)	(−0.14)	(−1.76)	(−1.43)
Market	0.014 *	0.015 **	0.001	0.000
	(1.95)	(2.09)	(0.20)	(0.08)
Cons	0.340 ***	0.298 ***	0.260 **	0.233 *
	(3.16)	(2.64)	(2.02)	(1.80)
Sigma_cons	0.151 ***	0.151 ***	0.137 ***	0.135 ***
	(46.43)	(46.43)	(43.93)	(43.93)
Industrial fixed effect	yes	yes	yes	yes
Year fixed effect	yes	yes	yes	yes
Observations	1183	1183	1065	1065
χ^2^-statistic	385.850	387.664	182.676	204.103

Note: * *p* < 0.10, ** *p* < 0.05, *** *p* < 0.01; t-values are in parentheses; The result of the difference between groups, chi2 = 2.73, *p* = 0.098.

**Table 7:** Grouping based on government influence-region.

Variables	Eastern Region (Low Government Influence)		Non-Eastern Region (High Government Influence)	
	(1)	(2)	(3)	(4)
CF	0.127 ***	0.131 ***	0.033 ***	0.051 ***
	(16.08)	(15.64)	(5.25)	(7.15)
AIR × CF		0.075		−0.231 ***
		(1.25)		(−5.06)
AIR		−0.050		0.027
		(−1.30)		(0.90)
Size	−0.023 ***	−0.023 ***	−0.007	−0.006
	(−4.86)	(−4.89)	(−1.63)	(−1.49)
Lev	0.115 ***	0.117 ***	0.034	0.029
	(3.40)	(3.44)	(1.16)	(0.97)
Cash	0.126 **	0.131 **	0.148 ***	0.157 ***
	(2.29)	(2.38)	(3.07)	(3.27)
Q	−0.001	−0.001	−0.001	−0.001
	(−0.20)	(−0.28)	(−0.33)	(−0.14)
Growth	−0.001	−0.001	−0.001	−0.000
	(−0.22)	(−0.28)	(−0.53)	(−0.12)
PPE	0.008	0.008	−0.015	−0.010
	(0.39)	(0.38)	(−0.58)	(−0.40)
First	−0.005	−0.003	0.032	0.026
	(−0.15)	(−0.10)	(1.02)	(0.84)
Dual	0.033 **	0.033 **	−0.001	−0.001
	(2.30)	(2.29)	(−0.04)	(−0.10)
State	−0.019	−0.019	0.003	0.003
	(−1.62)	(−1.56)	(0.30)	(0.28)
Age	−0.000	−0.000	−0.002 *	−0.002
	(−0.30)	(−0.20)	(−1.66)	(−1.56)
GDP	−0.012	−0.005	−0.009	−0.007
	(−1.03)	(−0.39)	(−1.05)	(−0.77)
Market	0.014 **	0.016 **	0.000	−0.001
	(1.98)	(2.22)	(0.07)	(−0.13)
Cons	0.394 ***	0.344 ***	0.169	0.141
	(3.60)	(3.02)	(1.31)	(1.10)
Sigma_cons	0.151 ***	0.151 ***	0.137 ***	0.135 ***
	(45.54)	(45.54)	(44.85)	(44.86)
Industry fixed effect	Yes	Yes	Yes	Yes
Year fixed effect	Yes	Yes	Yes	Yes
Observations	1140	1140	1108	1108
χ^2^-statistic	392.250	395.459	172.927	199.281

Note: * *p* < 0.10, ** *p* < 0.05, *** *p* < 0.01; t-values are in parentheses; The result of the difference between groups, chi2 = 3.55, *p* = 0.059.

**Table 8:** Grouping based on local formal environmental regulations.

Variables	Fewer Formal Environmental Institutions		More Formal Environmental Institutions	
	(1)	(2)	(3)	(4)
CF	0.083 ***	0.093 ***	0.055 ***	0.056 ***
	(11.34)	(12.78)	(8.07)	(8.15)
AIR × CF		−0.396 ***		−0.053
		(−7.15)		(−1.29)
Air		−0.052		0.037
		(−1.23)		(1.51)
Size	−0.011 **	−0.013 **	−0.008 **	−0.008 **
	(−2.08)	(−2.44)	(−2.20)	(−2.24)
Lev	0.087 **	0.076 **	0.040	0.039
	(2.18)	(1.97)	(1.59)	(1.52)
Cash	0.006	−0.033	0.123 ***	0.124 ***
	(0.08)	(−0.51)	(3.00)	(3.02)
Q	0.004	0.002	−0.001	−0.000
	(0.78)	(0.44)	(−0.24)	(−0.13)
Growth	0.001	0.001	−0.004	−0.004
	(0.09)	(0.13)	(−0.94)	(−0.97)
PPE	−0.084 **	−0.068 **	−0.041 **	−0.039 **
	(−2.53)	(−2.12)	(−2.29)	(−2.18)
First	−0.017	−0.012	0.007	0.004
	(−0.41)	(−0.30)	(0.28)	(0.16)
Dual	−0.018	−0.021	0.027 ***	0.027 ***
	(−1.08)	(−1.32)	(2.60)	(2.59)
State	0.010	0.010	−0.016 *	−0.017 *
	(0.67)	(0.72)	(−1.71)	(−1.82)
Age	−0.002	−0.001	−0.000	−0.000
	(−1.29)	(−1.09)	(−0.39)	(−0.30)
GDP	0.010	0.012	−0.021 ***	−0.024 ***
	(0.70)	(0.90)	(−2.75)	(−3.03)
Market	−0.003	−0.002	0.006 *	0.006 *
	(−0.76)	(−0.40)	(1.81)	(1.73)
Cons	0.113	0.110	0.238 ***	0.255 ***
	(0.79)	(0.79)	(2.72)	(2.90)
Sigma_cons	0.145 ***	0.140 ***	0.131 ***	0.131 ***
	(37.20)	(37.22)	(51.12)	(51.11)
Industry fixed effect	Yes	Yes	Yes	Yes
Year fixed effect	Yes	Yes	Yes	Yes
Observations	766	766	1427	1427
χ^2^-statistic	228.474	279.714	268.232	272.213

Note: * *p* < 0.10, ** *p* < 0.05, *** *p* < 0.01; t-values are in parentheses; The result of the difference between groups, χ^2^ = 4.91, *p* = 0.026.

**Table 9:** Robustness test using OLS and FE models.

Variables	OLS	FE
	(1)	(2)
CF	0.077 ***	0.070 ***
	(15.79)	(13.86)
AIR × CF	−0.223 ***	−0.224 ***
	(−6.91)	(−6.38)
AIR	0.031	0.073
	(1.42)	(1.01)
Size	−0.020 ***	−0.030 ***
	(−6.20)	(−3.05)
Lev	0.076 ***	−0.018
	(3.48)	(−0.50)
Cash	0.158 ***	0.275 ***
	(4.43)	(5.55)
Q	0.001	−0.001
	(0.41)	(−0.21)
Growth	0.000	−0.001
	(0.13)	(−0.35)
PPE	−0.029 *	−0.002
	(−1.82)	(−0.09)
First	0.010	0.056
	(0.45)	(0.98)
Dual	0.017 *	−0.010
	(1.88)	(−0.75)
State	−0.013 *	0.018
	(−1.69)	(0.84)
Age	−0.000	−0.026
	(−0.46)	(−0.61)
GDP	−0.017 ***	0.017
	(−2.58)	(0.53)
Market	0.003	−0.002
	(1.02)	(−0.22)
Cons	0.460 ***	0.534
	(6.03)	(1.58)
Industry fixed effect	Yes	
Year fixed effect	Yes	Yes
Firm fixed effect		Yes
Observations	2248	2248
*R^2^*	0.182	0.158
*F*-statistic	11.157	10.838

Note: * *p* < 0.10, *** *p* < 0.01; t-values are in parentheses.

**Table 10:** Endogeneity test with IV(2SLS) estimation.

Variables	First-Stage Regressions		Second-Stage Regressions
	AIR	AIR × CF	EPI
	(1)	(2)	(3)
PR	−0.003	−0.000	
	(−1.35)	(−0.53)	
RH	−0.064 ***	−0.009 *	
	(−4.81)	(−1.70)	
PR × CF	0.001	0.004 *	
	(0.14)	(1.87)	
RH × CF	−0.018	−0.148 ***	
	(−0.87)	(−10.90)	
CF	−0.001	0.032 ***	0.022 ***
	(−0.35)	(10.23)	(3.13)
AIR × CF			−0.290 *
			(−1.85)
AIR			−0.227
			(−1.11)
Size	0.002	−0.003 *	−0.014 ***
	(0.93)	(−1.62)	(−3.95)
Lev	0.065 ***	−0.000	0.075 ***
	(3.05)	(−0.02)	(2.77)
Cash	0.039	−0.002	0.180 ***
	(1.13)	(−0.11)	(4.62)
Q	0.003	−0.001	0.005 *
	(1.16)	(−0.61)	(1.75)
Growth	0.001	0.002 **	−0.000
	(0.40)	(2.14)	(−0.08)
PPE	−0.011	0.008	−0.046 ***
	(−0.71)	(0.84)	(−2.67)
First	0.108 ***	−0.010	0.041
	(4.95)	(−0.76)	(1.19)
Dual	−0.003	−0.005	0.018*
	(−0.44)	(−0.96)	(1.88)
State	0.0031 ***	−0.002	−0.010
	(4.07)	(−0.52)	(−0.98)
Age	0.000	0.000	−0.000
	(0.62)	(0.23)	(−0.32)
GDP	0.063 ***	0.000	0.004
	(9.84)	(0.19)	(0.27)
Market	0.015 ***	−0.002	0.003
	(5.89)	(−1.55)	(0.81)
Cons	−0.181 **	0.105*	0.237 *
	(−1.98)	(1.77)	(1.89)
Observations	2241	2241	2241
*F*-statistic	7.47	29.88	4.54

Note: * *p* < 0.10, ** *p* < 0.05, *** *p* < 0.01; t-values are in parentheses; the IV/2SLS model includes year and industry dummies; Anderson LM-statistic: chi2 = 27.864, *p* = 0.000; Cragg-Donald Wald F-statistic: 6.906; Sargan-statistic: chi2 = 7.679, *p* = 0.021.

**Table 11:** Regressions with PSM and alternative AIR variable.

Variables	PSM	Alternative AIR Variable
	(1)	(2)
CF	0.067 ***	0.013 *
	(9.15)	(1.83)
AIR × CF	−0.283 ***	
	(−7.34)	
AQI × CF		−0.001 ***
		(−3.09)
AIR	0.092 ***	0.000
	(4.42)	(0.04)
Size	−0.009 ***	0.000
	(−3.00)	(0.10)
Lev	0.038 *	0.070 **
	(1.76)	(2.40)
Cash	0.116 ***	0.042
	(3.38)	(0.81)
Q	−0.002	0.012 ***
	(−0.76)	(2.84)
Growth	0.000	−0.000
	(0.09)	(−0.14)
PPE	−0.008	−0.122 ***
	(−0.53)	(−4.29)
First	−0.022	−0.039
	(−1.01)	(−1.28)
Dual	0.020 ***	−0.006
	(2.58)	(−0.55)
State	−0.010	0.016
	(−1.23)	(1.58)
Age	−0.001	0.000
	(−0.92)	(0.51)
GDP	−0.021 ***	0.003
	(−3.24)	(0.34)
Market	0.005 *	−0.000
	(1.74)	(−0.14)
Cons	0.270 ***	−0.041
	(3.42)	(−0.34)
Sigma_cons	0.109 ***	0.105 ***
	(51.18)	(35.01)
Industry fixed effect	Yes	Yes
Year fixed effect	Yes	Yes
Observations	1438	673
χ^2^-statistic	244.478	178.434

Note: * *p* < 0.10, ** *p* < 0.05, *** *p* < 0.01; t-values are in parentheses.

**Table 12:** Univariate tests.

Variables	AIR_G = 0 (Regions with Poor Air Quality)		AIR_G = 1 (Regions with Non-Poor Air Quality)		Mean-Diff.	t-Test for Diff. in Means
	Obs	Mean	Obs	Mean		
EPI	1138	0.043	1110	0.046	−0.003	−0.551

**Table 13:** Mediating analysis.

Variables	Dependent Variable: DF	Dependent Variable: EPI	
	(1)	(2)	(3)
AIR	0.837 **	0.027	−0.007
	(1.98)	(1.18)	(−0.33)
AIR × CF		−0.232 ***	−0.063 **
		(−6.80)	(−2.10)
AIR × DF			−0.048 ***
			(−2.69)
CF		0.081 ***	0.027 ***
		(15.76)	(5.61)
DF			0.011 ***
			(12.36)
Size	0.041	−0.016 ***	0.000
	(0.59)	(−4.81)	(0.10)
Lev	3.545 ***	0.082 ***	0.070 **
	(7.59)	(3.52)	(2.40)
Cash	−5.453 ***	0.185 ***	0.042 *
	(−6.08)	(4.90)	(1.81)
Q	−0.105	0.002	0.012 ***
	(−1.53)	(0.50)	(2.84)
Growth	0.048	0.000	−0.000
	(0.97)	(0.13)	(−0.14)
PPE	−6.019 ***	−0.023	−0.122 ***
	(−11.97)	(−1.36)	(−4.29)
First	0.583	0.018	−0.039
	(1.15)	(0.75)	(−1.28)
Dual	0.303	0.017 *	−0.006
	(1.54)	(1.80)	(−0.55)
State	−0.148	−0.008	0.016
	(−0.84)	(−0.99)	(1.58)
Age	−0.003	−0.001	0.000
	(−0.22)	(−0.84)	(0.51)
GDP	−0.670 ***	−0.016 **	0.003
	(−4.46)	(−2.28)	(0.34)
Market	0.278 ***	0.004	−0.000
	(4.56)	(1.34)	(−0.14)
Cons	5.202 ***	0.341 ***	0.168 **
	(3.02)	(4.21)	(2.19)
Sigma_cons	3.527 ***	0.152 ***	0.156 ***
	(71.67)	(63.93)	(70.71)
Industry fixed effect	Yes	yes	Yes
Year fixed effect	Yes	yes	Yes
Observations	2248	2248	2248
χ^2^-statistic	966.793	392.904	415.207

Note: * *p* < 0.10, ** *p* < 0.05, *** *p* < 0.01; t-values are in parentheses.

## Data Availability

The datasets used and analyzed during the current study are available from the corresponding author on reasonable request.
